# A new titanium-covered transobturator tape for surgical treatment of stress urinary incontinence

**DOI:** 10.1007/s00192-021-04976-8

**Published:** 2021-10-02

**Authors:** Anne-Claude Fahrni, Cornelia Betschart, Jean Bouquet de la Jolinière, Jean-Bernard Dubuisson, Anis Feki, Attila Louis Major

**Affiliations:** 1 University & Department of Obstetrics and Gynecology, Cantonal Hospital, 1708 Fribourg, Fribourg Switzerland; 2 Department of Gynecology, University Hospital Zurich, University Zurich, Frauenklinikstrasse 10, 8091 Zurich, Switzerland; 3 Femina Gynecology Center, Rue Emile-Yung 1, 1205 Geneva, Switzerland

**Keywords:** Titanium-covered transobturator tape, Midurethral slings, Stress urinary incontinence, Incontinence outcome questionnaire (IOQ), Patient satisfaction

## Abstract

**Introduction and hypothesis:**

To assess the long-term satisfaction, cure rate and safety of a new titanium-covered transobturator tape compared to polypropylene tape for the treatment of stress urinary incontinence (SUI).

**Methods:**

A prospective study was conducted with 151 patients. Seventy patients underwent transobturator sling surgery with titanium tape from 2011 to 2019, and a historical control group (CG) of 81 patients was treated with a noncoated tape and underwent incontinence surgery from 1999 to 2009. We compared patient-reported outcome measures (PROMs) with the incontinence outcome questionnaire (IOQ).

**Results:**

The median follow-up was 2½ years in both groups. Based on responses to the IOQ, a statistically significantly shorter time of recovery (IOQ 15: 21.3 ± 26.4 [TG], 40.2 ± 38.5 [CG], *p* = 0.02), improvement of sex life (IOQ 13: 34.1 ± 29.4 [TG] vs. 65.3 ± 35.6 [CG], *p* = 0.01) and less voiding dysfunction (IOQ 19: 30.9 ± 28.1 [CG], 9.3 ± 18.6 [TG], p = 0.01) were observed in the TG. Objectively, no postoperative urinary retention was observed in the TG, but four cases were described in the CG. Ten patients needed a reoperation for SUI in the CG compared to three in the TG (*p* = 0.03).

**Conclusion:**

The titanium-covered transobturator sling had superior recovery time, improved sexual function and reduced reoperation rate compared to a historical polypropylene group.

## Introduction

Stress urinary incontinence (SUI) is a common and underdiagnosed condition, affecting 4–35% of women [1, 2]. SUI treatment encompasses both conservative and surgical therapies. Nowadays, the insertion of midurethral slings is the gold standard surgical treatment for SUI. The sling provides tension-free support for the midurethra, and when there is pressure on the bladder, the immobile tape compresses the urethra and prevents urinary leakage. Surgery can be proposed to the patient either after unsuccessful conservative treatment or as a first-line treatment for symptomatic or occult SUI in cases of pelvic organ prolapse [3]. Conservative therapy includes behavioural modification (weight loss, dietary changes, smoking cessation), the elimination of contributory factors, pelvic floor muscle exercises, bladder training, vaginal pessaries or medication (duloxetine, local oestrogen [4–6]). Acute urinary infection and ongoing pregnancy are contraindications to the insertion of midurethral slings.

Before midurethral sling surgery, it is important to counsel the patient about the different types of slings and procedural techniques available. To decrease the risk of bladder injury, the transobturator tape (TOT) route was created in 2001. In this procedure, the slings are introduced through the two obturator foramens [7]. This technique can be executed in two directions: the inside-out variation (TOT-I), which is accomplished by inserting the trocars from a midurethral vaginal incision to exit through bilateral groin incisions, and the outside-in variation (TOT-O). Evidence suggests that these two methods are equally effective with comparable long-term efficacies and similarly low complication rates [8].

Although both TVT and TOT have equivalent subjective cure rates, they differ in terms of efficacy and adverse effects. TOT is the preferred method when important adhesions of the bladder or intestines are expected. TOT is also best suited for obese women or women with bleeding diathesis [9, 10]. However, a higher rate of sling erosion was observed with TOT than with TVT [11]. Therefore, it is important to find new strategies and sling materials to decrease the exposition rate. Many studies have compared the different surgical techniques for the placement of suburethral slings in terms of efficacy, side effects and relapse, but few studies have compared different mesh compositions. Although the current meshes are made from synthetic macroporous monofilament polypropylene and are 10 mm wide, there are many variations on the market. Titanium is known for its good biocompatibility (less inflammation and fewer foreign body reactions) and is used to manufacture various medical prosthetic devices [12]. This material has been proven to be safe in recent studies and seems to be superior to conventional mesh. For example, titanised transobturator slings provided favourable midterm continence outcomes in male patients with SUI [13].

In this context, it is appropriate to compare the titanium-coated sling with the conventional one. The primary objective of this study was to compare the long-term satisfaction and cure rates of a new, titanium-covered transobturator tape and common polypropylene tape for the treatment of SUI. The secondary objective was to analyse the side effects and complications of the two different tapes.

## Materials and methods

**Fig. 1:**
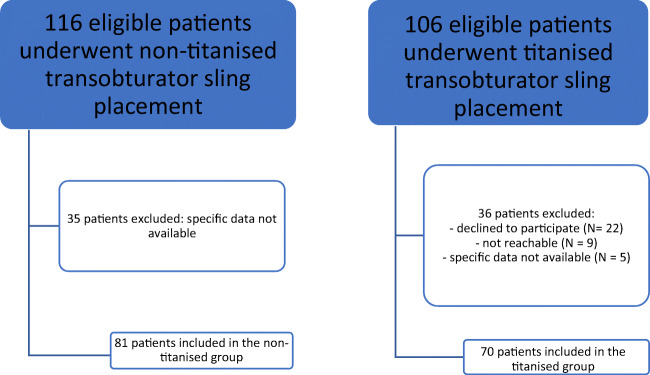
Flowchart

A prospective single-arm trial was conducted at the Fribourg Cantonal Hospital. The subjects included 106 women who had undergone TOT surgery using a titanium-covered tape from November 2011 to November 2019. The control group (CG) consisted of a historical cohort of 116 patients who underwent insertion of non-titanised polypropylene tapes and responded to the incontinence outcome questionnaire (IOQ) between 1999 and 2007 at the University Hospital of Zurich. The IOQ, validated for the German language, is considered as a valid and reliable instrument for assessing QOL and patient-reported outcomes after insertion of a midurethral tape when baseline or preoperative data are unavailable [14]. Patients in the titanium group (TG) were invited to participate in the study through a written letter sent at least 6 months after the operation. After the patients consented, their preoperative subjective and urodynamic parameters were collected. For postoperative data, an interview was conducted using the IOQ. Patients who were not reachable, whose medical data of interest were not obtainable or who declined to participate were excluded and did not complete the questionnaire (35 patients in the CG versus 36 patients in the TG) (Fig.1). The procedures and questionnaire used for the CG were the same as those used in another large study, published in 2012, that examined different tapes in Switzerland [15].

Demographic and urodynamic parameters were collected and saved electronically after patients gave written informed consent. Patients with insufficient data were excluded.

Subjective data and cure rates were assessed using the IOQ. The survey consisted of 27 questions divided into six parts: (1) four questions related to symptoms (pain, postoperative symptoms, preoperative overactive bladder [OAB] symptoms and change in OAB symptoms pre- and postoperatively); (2) four questions about complications (urinary or other infections, hospital readmission and residual urine); (3) seven questions about quality of life (QoL) (felt tired/drained/lacking energy, felt irritable/snappy, felt depressed/tearful, general evaluation of health, limitations in daily activities, change in sexual activity and change in body perception); (4) five questions about satisfaction (changes of symptoms pre- and postoperatively, time of recovery, satisfaction with information, improvement in well-being and recommending the operation); (5) one question about problems with urinary incontinence before surgery; (6) six questions related to demographic and treatment-related information (age, occupation, living arrangements, reason for operation, hormone replacement therapy [HRT] and time interval to operation).

Each item was rated on a scale from 0 to 100 (0 = minimum, 100 = maximum impairment) from which the satisfaction extended score was calculated. This score corresponded to the mean of the 15 questions (IOQ5, IOQ6, IOQ7, IOQ 9, IOQ10, IOQ11, IOQ12, IOQ13, IOQ14, IOQ15, IOQ16, IOQ17, IOQ18, IOQ19 and IOQ21) about QoL, satisfaction, postoperative symptoms, postvoid residual urine volume and change in OAB symptoms.

Menopausal status was determined from the response to question 26 of the IOQ concerning age (from 51 years of age), the use of HRT and posthysterectomy status. Sphincter insufficiency was defined as a maximum urethral closure pressure < 20 cmH_2_O.

In the TG, an inserted titanium-covered vaginal tape with the brand name TiLOOP® Tape, manufactured and distributed by pfm medical, Germany, was used for both routes (TOT-O [outside-in] and TOT-I [inside-out]). The classical noncoated polypropylene vaginal tape for TOT-I, which was used in the CG, was manufactured by Ethicon, Neuchâtel, Switzerland, and distributed by Gynecare, Switzerland. The TOT-O tape Monarc® was manufactured by American Medical Systems, Minnetonka, MN, USA, and distributed by Promedics, Switzerland.

In both groups, the two techniques (TOT-O and TOT-I) were used depending on the surgeon’s preference. The minimum follow-up was 6 months. Special care was taken in cleaning before and during surgery as this may play a key role in whether implants such as tapes are rejected. Betadine Aqueous solution was used for cleaning the vagina and alcoholic solution for the vulva and the inguinal region. Before inserting the titanium tape, new sterile gloves were put on.

Ethical approval was obtained from the Cantonal Commission for Ethics in Human Research (CER-VD; Project-ID: 2019–00705).

Statistical evaluation was carried out using IBM SPSS Statistics 27. Continuous data are presented as mean ± standard deviation. At baseline, the groups were compared using independent sample *t*-tests for normally distributed values and Fisher’s exact test for dichotomous variables. To compare both groups (TG and CG), the Mann-Whitney test was performed using the score obtained from the questionnaire. Since ages were statistically different between the groups and presented an important bias in testing the TG against the CG, we aligned the parameters for this covariable. A multiple linear regression analysis and an ANCOVA (analysis of covariance) test were also performed to study the impact of different variables on the IOQ extended score and to analyse the risk factor for complications. The age disparity was eliminated using an ANCOVA test to make the groups comparable. Two-sided*p*-values < 0.05 were considered statistically significant.

## Results

A total of 151 patients were eligible: 70 (46%) in the TG and 81 (54%) in the CG (Fig.1).

The baseline characteristics of the patients are summarized in Table 1. Most patients in the CG were > 60 years old and postmenopausal, which is associated with a lower success rate in incontinence operations [16]. Age, menopausal status and BMI were significantly different between the two groups. Unlike the age discrepancy, the difference in menopausal status (TG: 34/70 [48%] postmenopausal, CG: 73/79 [92%] postmenopausal) and BMI (TG: 29.2 ± 0.8, CG: 26.5 ± 0.5) between the groups did not significantly influence the results as demonstrated by the linear regression analysis.

**Table.1:** Baseline characteristics of the 151 patients that qualified for the study. Data are expressed as mean ± standard deviation or number of patients (percentage)

	Tape with titanium *N* = 70	Tape without titanium *N* = 81	*P* value (Mann-Whitney test)
Age at operation (years)	50.9 ± 1.2	63.8 ± 1.2	0.00 ^a*^
Body mass index (kg/m^2^)	*N* = 70 29.2 ± 0.8	*N* = 79 26.5 ± 0.5	0.01 ^a*^
Parity	2.4 ± 0.1	2.0 ± 0.1	0.31 ^a^
History of smoking	17 (24.3)	19 (23.5)	1.00 ^b^
Hormonal status Premenopausal Postmenopausal with HRT Postmenopausal without HRT	*N* = 70 36 (51.4) 10 (14.3) 24 (34.3)	*N* = 79 7 (8.8) 27 (34.2) 45 (57)	0.00 ^b*^
Previous hysterectomy	15 (21.4)	28 (34.6)	0.10
Previous incontinence surgery	*N* = 70 2 (2.8)	*N* = 81 7 (8.6)	0.18 ^b^
Sling insertion	1	1	
Abdominal or vaginal colposuspension	1	5	
Intravesical botulinum toxin	0	1	
Type of urinary incontinence			0.39 ^b^
Mixed	48 (68.6)	50 (61.7)	
Pure stress	22 (31.4)	31 (38.3)	
Type of surgery			0.05 ^b^
TOT-O (outside-in)	38 (54.3)	47 (58)	
TOT-I (inside-out)	32 (45.7)	24 (42)	
Concomitant hysterectomy	10 (14.3)	12 (14.8)	1.00
Concomitant prolapse surgery	*N* = 70 14 ^$^ (20)	*N* = 80 15 ^§^ (18.8)	1.00 ^b^
Anterior colporrhaphy	5	15	
Posterior colporrhaphy	9	10	
Vaginal colposuspension	2	0	
Sacrospinous ligament fixation (Richter)	0	2	
Laparoscopic latero-suspension (Kapandji-Dubuisson)	1	0	

*Statistically significant (*p* < 0.05)

^a^Mann-Whitney

^b^Fisher’s exact test

SD = standard deviation, HRT = hormone replacement therapy

^$^Three patients underwent hysterectomy and posterior colporrhaphy simultaneously, two underwent hysterectomy with vaginal colposuspension, two underwent anterior and posterior colporrhaphy, one underwent hysterectomy with anterior and posterior colporrhaphy and one underwent anterior and posterior colporrhaphy with laterosuspension

^§^One patient underwent hysterectomy and anterior colporrhaphy simultaneously, one underwent anterior and posterior colporrhaphy, seven underwent hysterectomy with anterior and posterior colporrhaphy, one underwent hysterectomy with sacrospinous ligament fixation and posterior colporrhaphy and one underwent hysterectomy with anterior and posterior colporrhaphy and sacrospinous ligament fixation

The groups were comparable for the remaining features. Only one patient in each group had undergone a sling insertion in the past. The patient in the TG had undergone a hysterectomy with concomitant TOT-I with a noncoated tape 4 years earlier. She suffered from a persistence of predominant stress mixed urinary incontinence and benefited from a second TOT-I with a titanised sling, which was a success. In the CG, a patient had undergone a hysterectomy with concomitant TVT 4 years earlier, which became complicated 1 year after surgery by chronic urinary retention and new urge incontinence that required sling revision. The two surgical techniques (TOT-O and TOT-I) were used in equal proportions in the two groups.

The urologic evaluation showed that stress incontinence was predominant in most patients suffering from mixed urinary incontinence in both groups. Preoperative urodynamic data were available for 114 patients, and the data were expressed as means and standard deviations. The MUCP (maximum urethral closure pressure) was lower in the CG, which had the older patients (TG: 62.4 ± 21.4, CG: 39.3 ± 20.1 cmH_2_O), and water-filled catheters were used for patients in this group. However, the technique used for pressure measurement (air-charged catheter in the TG vs. water-charged catheter in the CG) was different between the groups, and this prevented a statistical comparison of these values [17]. No patient suffered from a sphincter insufficiency in the TG; however, 8 of the 47 eligible patients in the CG had sphincter insufficiency. The measure of the volume at first desire to void was in the normal range in each group but was significantly lower in the TG even after adjusting for age (TG: 174.5 ± 82, CG: 252.5 ± 111 ml, *p* = 0.03).

At the time of completion of the IOQ, the median follow-up period was 2.5 years in both groups. Based on responses to the IOQ, the subjective efficiency of the sling was 83% in the TG and 80% in the CG.

After adjustment for age, the following outcomes were statistically different between the groups (Table 2): More preoperative OAB symptoms were described in the CG (IOQ 20: 64.3 ± 48.3 [TG], 81.5 ± 39.1 [CG], *p* = 0.01). A shorter time of recovery (IOQ 15: 21.3 ± 26.4 [TG], 40.2 ± 38.5 [CG], *p* = 0.02), improvement of sex life (IOQ 13: 34.1 ± 29.4 [TG], 65.3 ± 35.6 [CG], *p* = 0.01) and better satisfaction with information (IOQ16: 46.1 ± 19.8 [TG], 54.3 ± 21.2 [CG], *p* = 0.03) were observed in the TG.

**Table.2:** Postoperative subjective long-term results. Data are expressed as mean ± standard deviation (range)

	Tape with titanium *N* = 70	Tape without titanium *N* = 81	*P* value (Mann-Whitney test)	*P* value (ANCOVA)
Symptoms				
Pain (IOQ1)	11.4 ± 22.5	7.21 ± 15.6	0.45	0.14
Symptoms preoperative (IOQ8)	71.8 ± 25.8	74.4 ± 26.5	0.44	0.36
Symptoms postoperative (IOQ9)	16.1 ± 19.5	19.1 ± 29.1	0.68	0.69
OAB preoperative (IOQ20)	64.3 ± 48.3	81.5 ± 39.1	0.17	0.01*
Change in OAB symptoms pre- or postoperatively (IOQ21)	26.9 ± 27.6	24.2 ± 27.7	0.56	0.37
Complications				
Urinary infection (IOQ2)	17.1 ± 38	33.3 ± 47.7	0.24	0.10
Other infection (IOQ3)	17.1 ± 37.9	16.1 ± 36.9	0.85	0.10
Hospital readmission (IOQ4)	1.4 ± 11.9	12.4 ± 33.1	0.03 *	0.09
Residual urine (IOQ19)	9.3 ± 18.6	30.9 ± 28.1	0.00 *	0.01 *
Quality of life				
Felt tired/drained/lacking (IOQ5)	40 ± 26.4	34.6 ± 28.1	0.21	0.11
Felt irritable/snappy (IOQ6)	38.6 ± 28.5	35.2 ± 27.9	0.47	0.32
Felt depressed/tearful (IOQ7)	33.6 ± 29.1	36.4 ± 30.6	0.59	0.71
Global evaluation of health (IOQ10)	34.6 ± 24.2	45.3 ± 24.8	0.12	0.07
Limitations in daily activities (IOQ12)	27.9 ± 33.7	34.6 ± 32.3	0.16	0.91
Change in sex life (IOQ13)	34.1 ± 29.4	65.3 ± 35.6	0.00 *	0.01 *
Change in feeling about body (IOQ14)	22.5 ± 34.6	28.4 ± 33.5	0.18	1.00
Satisfaction				
Symptom changes pre- and postoperatively (IOQ11)	18.4 ± 26.4	17.3 ± 31.5	0.65	0.96
Time of recovery (IOQ15)	21.3 ± 26.4	40.2 ± 38.5	0.03 *	0.02 *
Satisfaction with information (IOQ16)	46.1 ± 19.8	54.3 ± 21.2	0.01 *	0.03 *
Improvement in well-being (IOQ17)	21.4 ± 28.3	21 ± 31	0.69	0.24
Recommend operation (IOQ18)	12.8 ± 25.4	20.1 ± 33.1	0.18	0.35
IOQ QoL extended score (0–100)	27.1 ± 18.9	34.2 ± 18.5	0.03 *	0.33

*Statistically significant (*p* < 0.05)

The evaluation of the responses about complications revealed a significantly higher rate of reported voiding dysfunction in the CG compared to the TG (IOQ 19: 30.9 ± 28.1 [CG], 9.3 ± 18.6 [TG], *p* = 0.01). Objectively, no postoperative urinary retention was observed in the TG, but four cases (4–9%) were described in the CG.

No difference was found between the TG and the CG for the remaining items of the questionnaire and for satisfaction in changing the quality of life after adjustment for age (IOQ extended score, Table 2).

Objectively, ten patients needed a second surgery in the CG as opposed to three in the TG, and this was statistically significant even when age was accounted for (*p* = 0.03, ANCOVA). Partial tape excision due to tape exposition was performed in one patient in the TG compared to three patients in the CG, among whom one underwent a second tape placement. There was no statistically significant difference in exposure of the sling between both groups. One case of vaginal tape exposure (1.4%) was found in the TG 3 years after sling placement compared to three (3.7%) in the CG, appearing between 2 and 18 months after surgery. In the CG, one patient had a partial tape excision because of chronic urinary retention and two had complete tape incision because of bladder outlet obstruction. Owing to the recurrence of SUI, two patients in the TG and four patients in the CG received a second tape. One patient in the CG had an intravesical injection of botulinum toxin. Regarding risk factors for procedure failure or recurrence out of these ten patients from the CG, eight were > 50 years old, one had a BMI > 35 kg/m², two suffered from sphincter insufficiency, two had concomitant prolapse surgery and eight suffered from mixed incontinence. In the TG, only three patients required a second operation, and among these three, one was > 50 years old, one had mixed incontinence and one was diabetic. The complementary multivariate analysis for the influence of the potential risk factors on recurrence needing a reoperation in the two groups showed that the presence of mixed incontinence was the most important risk for recurrence (*p* < 0.01) followed by the use of non-titanium tape (*p* = 0.02). The other risk factors showed no significant correlations. Additional risk factors for recurrent or persistent urinary incontinence after primary surgical treatment are an increasing stage of pelvic organ prolapse (POP) and obstructive voiding symptoms [18]. In this study, no patient was diagnosed with obstructive voiding dysfunction, and the patients with concomitant prolapse surgery had more advanced stages of pelvic organ prolapse.

The rates of postoperative complications, such as new or worsened urgency incontinence and postoperative pain, were equal between the two groups. New or worsened urgency incontinence was reported by five patients (7.1%) in the TG and eight patients (9.9%) in the CG. Only one patient in the TG reported chronic groin pain. Nine patients (11.1%) in the CG and 13 (18.3%) in the TG reported temporary unspecific pain. Finally, we noted that 68% of postoperative complications were in the TOT-O group compared to 32% in the TOT-I.

## Discussion

To our knowledge, this is the first study comparing outcomes of titanium-covered mesh tape to those of uncovered polypropylene mesh tape in women. The results are consistent with those of similar studies about titanium tape use in men [13].

First, significantly more severe preoperative OAB symptoms were observed in the CG, potentially explained by a more important volume at first desire to void and by the higher age found in the CG (Table 2). Indeed, the sensation of the bladder declines with age, increasing the volume at desire to void. Therefore, the greater the volume at the time someone needs to urinate, the shorter the warning period before a urine leakage occurs [20].

Secondly, significantly more patients underwent a second surgery for persistent or recurrent incontinence in the CG (Table 1). Interestingly, age > 50 years, severe obesity, urethral incompetence, concomitant prolapse surgery and urgency urinary incontinence are risk factors for the recurrence of incontinence and at least one of those were present in these patients [19–21]. In cases of sphincter insufficiency or recurrence of incontinence after primary surgery, the literature reports that the retropubic route should be preferred [22].

The most common complication reported by the patients was voiding dysfunction (IOQ 19). Objectively, only four patients (4.9%) in the CG suffered from urinary retention postoperatively, which is comparable to the rate of 4–11% of postoperative dysuria and urinary retention after TOT insertion reported in the literature [24, 27]. In contrast, no postoperative urinary retention was observed in the TG. Two reasons can be given for the large discrepancy between subjective and objective outcomes regarding voiding dysfunction. The first is that mild symptoms are not observed in a urodynamic examination. Another probable reason is the time interval between the urodynamic examination and the implementation of the questionnaire.

On average, patients in the TG reported postoperative better sex lives compared to those in the CG. All patients who had vaginal exposure of the sling reported a deterioration in sex life. Our results are consistent with those from a meta-analysis performed by Ford et al. [9] that reported 1–9% dyspareunia after transobturator sling insertion and 2.4% postoperative vaginal exposure. In animal studies, a lightweight titanium-coated mesh demonstrated superior biocompatibility in terms of chronic inflammatory reactions without a loss of critical tensile strength compared with noncoated meshes [15]. In a prospective case-controlled study by Wang et al. [23], a potential immunologic reaction of the vagina to polypropylene mesh was found after the removal of the prosthesis and analysis of the surrounding tissue in cases of vaginal exposure. This finding of host versus prosthesis reaction and inflammation could be an argument in favour of a material with a potentially better compatibility such as titanium. According to the risk factors for vaginal exposure, the complementary multivariate analysis in this study demonstrated that smoking and advanced age significantly increased the risk of vaginal mesh exposure (*p* = 0.03).

Other postoperative complications reported were comparable between the two groups and our results reflect those in the literature. Indeed, new or worsened urgency incontinence is reported in 9% of cases [24]. The frequency of postsurgical groin pain is estimated between 12 and 16% [25, 26].

This study is limited by its retrospective design with a historical cohort. The fact that the control group was operated on 10 years earlier is a weakness of our study. Therefore, there may be a learning error as the first cases have been shown to have higher complication rates. The heterogeneity between the TG and CG in terms of age, BMI, postmenopausal status, HRT and surgeons is a possible confounder. The most significant confounding factor for the IOQ score between both groups was the age of the participants. Therefore, we matched the TG and the CG for the age with the ANCOVA analysis to obtain more comparable data and more accurate results. Although the technique of insertion was not a confounder for the IOQ extended score, the different complication rates between both techniques could suggest that complications are more related to the insertion technique than the type of tape used, which is another possible bias in our study. However, this contrasts with the results of the longest randomized study follow-up comparing the inside-out and outside-in procedures, which demonstrated comparable long-term efficacy with low complication rates [8]. Another possible bias is the timing between surgery and completion of the questionnaire, which was sometimes longer than the recommended 3 months. Another limitation of our study is the reliability and validity of the IOQ based only on studies in an Austrian population, and the method of choice for QOL assessment still needs to be evaluated with validated questionnaires both before and after treatment.

One of the strengths of this study is its alignment with a recent statement by the CHORUS (International Collaboration for Harmonizing Outcomes, Research, and Standards in Urogynaecology and Women’s Health) group that proposed an interim use of three main outcome domains: treatment success rate, urodynamic evaluation outcomes, such as OAB symptoms and voiding dysfunction, and patient-reported outcome measures on QoL, such as sexual function and global patient satisfaction with the use of validated questionnaires [27]. Complications and reoperations were also stated accurately. Other strengths are the sample size, long uniform duration of follow-up and use of the same questionnaire on a similar population of patients after age alignment.

In conclusion, the titanium-covered transobturator slings were superior to noncoated tapes used in a historical group, promoting less voiding dysfunction, faster recovery, potential amelioration of sex life and fewer reoperations. However, further studies are needed to draw definitive conclusions.

## Abbreviations

CG: Control group
CHORUS: International Collaboration for Harmonizing Outcomes, Research, and Standards in Urogynaecology and Women’s Health
HRT: Hormone replacement therapy
IOQ: Incontinence outcome questionnaire
QOL: Quality of life
MUCP: Maximum urethral closure pressure
OAB: Overactive bladder
POP: Pelvic organ prolapse
SUI: Stress urinary incontinence
TG: Titanium group
TVT: Tension-free vaginal tape
TOT: Transobturator tape
TOT-I: Transobturator tape inside-out technique
TOT-O: Transobturator tape outside-in technique
